# Role of the fronto-parietal cortex in prospective action judgments

**DOI:** 10.1038/s41598-021-86719-9

**Published:** 2021-04-02

**Authors:** Laurie Geers, Mauro Pesenti, Gerard Derosiere, Julie Duque, Laurence Dricot, Michael Andres

**Affiliations:** 1 Psychological Sciences Research Institute, Université catholique de Louvain, Place Cardinal Mercier 10, Louvain-la-Neuve, Belgium; 2 Institute of Neuroscience, Université catholique de Louvain, Avenue Mounier 53, Brussels, Belgium

**Keywords:** Cognitive neuroscience, Human behaviour

## Abstract

Prospective judgments about one’s capability to perform an action are assumed to involve mental simulation of the action. Previous studies of motor imagery suggest this simulation is supported by a large fronto-parietal network including the motor system. Experiment 1 used fMRI to assess the contribution of this fronto-parietal network to judgments about one’s capacity to grasp objects of different sizes between index and thumb. The neural network underlying prospective graspability judgments overlapped the fronto-parietal network involved in explicit motor imagery of grasping. However, shared areas were located in the right hemisphere, outside the motor cortex, and were also activated during perceptual length judgments, suggesting a contribution to object size estimate rather than motor simulation. Experiment 2 used TMS over the motor cortex to probe transient excitability changes undetected with fMRI. Results show that graspability judgments elicited a selective increase of excitability in the thumb and index muscles, which was maximal before the object display and intermediate during the judgment. Together, these findings suggest that prospective action judgments do not rely on the motor system to simulate the action per se but to refresh the memory of one’s maximal grip aperture and facilitate its comparison with object size in right fronto-parietal areas.

## Introduction

The capacity to imagine the progression of an action, before it is eventually executed, is a fundamental aspect of adaptive behavior. For example, anticipating a failure to grasp an object because it is too large will enable the implementation of motor adaptations facilitating the action, such as using two hands. It is commonly assumed that such prospective action judgments require the actor to compare the characteristics of the object to their body capabilities^1^. In the particular case of graspability judgments (i.e., judging whether one would be capable to grasp an object), this implies to compare object size to maximal achievable grip aperture. In a seminal paper^2^, Johnson went one step further by assuming that this comparison is preceded by the mental simulation of the reach and grasp movement. This simulation would recruit the same processes as those involved in action planning and execution, namely encoding the visual properties of the object (e.g., the size and orientation), activating the sensorimotor representation of the effectors (e.g., the hand), and computing the visuo-motor transformations required to achieve the action given a set of biomechanical rules^2–4^. This view has been endorsed by other authors and has become very popular with the emergence of embodied accounts of cognitive judgments^5–7^.

Results of functional magnetic resonance imaging (fMRI) studies are often taken as evidence that prospective action judgments involve motor simulation^8,9^. Existing data indeed suggest that prospective action judgments involve the same fronto-parietal regions as those involved in explicit simulation or execution of an action, including areas within the anterior part of the intraparietal sulcus [aIPS], the ventral and dorsal premotor cortex [PMv, PMd], and the dorsolateral prefrontal cortex^8,10–13^. However, the precise overlap between prospective action judgments and motor imagery has never been directly measured in previous fMRI studies, leaving the question of a possible common neural substrate unsolved. Moreover, some regions within this fronto-parietal network (in particular the aIPS) have also been associated with magnitude processing in non-motor tasks, such as length, size, or number comparison^14–16^. The general relevance of magnitude information for action may in fact explain why magnitudes as varied as distance, size, or duration are integrated within a generalized magnitude system located in the parietal cortex^17^. Making prospective judgments about an object-directed action and mentally simulating the action both require the computation of magnitude estimates, such as grip aperture or reaching distance, but existing studies have left out the possibility that the fronto-parietal areas activated during prospective action judgments and motor imagery might actually reflect magnitude processing. Finally, when considering specifically the primary somatosensory (S1) or motor (M1) cortices, an increase of the blood-oxygen-level-dependent (BOLD) signal has been occasionally reported during explicit simulation of an action but not during prospective action judgments^12^. Hence, the fronto-parietal activations found during prospective action judgments might in fact reflect a direct comparison between object size/distance and grip/arm representation, rather than some form of motor simulation, as assumed in earlier definitions of this process^1^.

In order to test whether or not prospective action judgments involve motor simulation, we exploited the respective advantages of fMRI, as a method to record and compare the whole-brain BOLD response, and transcranial magnetic stimulation (TMS), as a method to probe corticospinal excitability (CSE) modulation to hand muscles. In the first experiment, we used fMRI to map neural activity in the whole brain while participants were making judgments about their ability to grasp rectangles of various lengths between their index finger and thumb (i.e., graspability judgments) or while they were explicitly imagining themselves grasping various objects (i.e., motor imagery). In order to differentiate the brain areas involved in action processing from those involved in magnitude processing, a control task required participants to judge whether rectangles were larger than a perceptual reference (i.e., length judgments). Under the *motor simulation* hypothesis, graspability judgments and motor imagery should show overlapping activations within the fronto-parietal areas beyond the level of activation measured during length judgments. Under the *comparison-without-simulation* hypothesis, we expect the shared network to be equally activated during length judgments because its role would be to serve magnitude processing, irrespective of body capabilities. To gain further knowledge into the spatial distribution of activations within the areas shared by motor imagery, graspability and length judgments^13^, we computed voxelwise correlations between the patterns of activations elicited by each task^18,19^. In a second experiment, we directly measured motor excitability changes in a set of hand muscles by means of motor-evoked potentials (MEPs) induced by TMS during graspability and length judgments. A close look at the available data indicates that, compared to action execution, motor imagery only induces a 50–70% rise in BOLD signal in M1^20,21^. This might cause M1 activations to remain undetected in most studies, though laminar fMRI has recently proved efficient in detecting reliable activations in the superficial layer of M1 during motor imagery^22^. An alternative is to investigate the instantaneous modulation of the CSE excitability of hand muscles, during motor imagery, through the recording of MEPs induced by single pulse TMS over M1 in the contralateral hand^23^. MEPs provide a sensitive output measure of fronto-parietal activity during motor imagery, as CSE changes may be caused by M1 or any frontal or parietal area remotely connected to M1^24^. It is also possible to track the unfolding of the imagined movement by delivering TMS pulses at different times relative to the task onset^25^. Finally, when TMS is used to excite pools of corticospinal cells projecting onto different muscles, CSE changes are specific to the muscles involved in the imagined movement^25^. Under the *motor simulation* hypothesis, graspability judgments, but not length judgments, should be associated with an increase of MEPs in the first dorsal interosseus (FDI) and the abductor pollicis brevis (APB), which both contribute to adjust the precision grip, but not in the abductor digiti minimi (ADM), which is not relevant for thumb-index pinch movements. Moreover, TMS should elicit larger MEPs when it is delivered as the imagined grasp movement unfolds. Under the *comparison-without-simulation* hypothesis, no difference in MEP amplitude is expected during graspability and length judgments since the comparison of object size with grip aperture is analogue to the comparison of two lengths.

## Methods

### fMRI experiment

#### Participants

Thirty-two healthy volunteers (21 females, mean age and standard error (SE): 23 ± 0.41 years) participated in this fMRI experiment. They were all right-handed according to the Edinburgh inventory questionnaire^26^, had no history of neurological or psychiatric disorders, had normal or corrected-to-normal vision, and were unaware of the purpose of the study. They gave their written informed consent prior to the experiment. The study was non-invasive, performed in accordance with the ethical standards of the Declaration of Helsinki, and approved by the Biomedical Ethical Committee of the Université catholique de Louvain. The data of one participant were excluded because of drifts superior to 5 mm in translation in the functional images and those of another participant because unusually strong susceptibility artifacts were visible at the level of the frontal cortex. The analyses were conducted on the 30 remaining participants.

#### Tasks and stimuli

**Figure 1:**
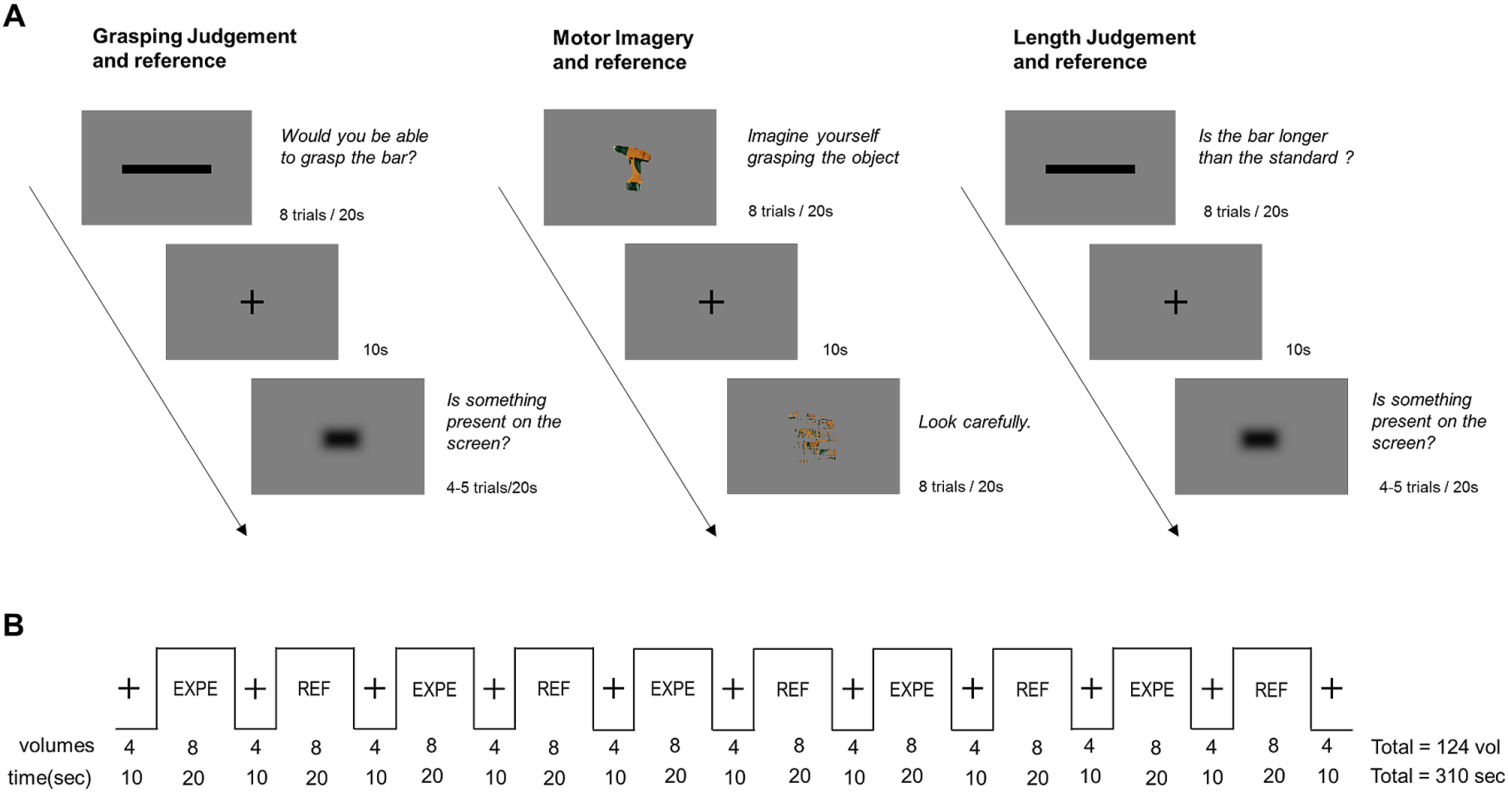
Schematic representation of the time course of the fMRI experiment. Part (**A**) represent the experimental tasks and their reference with an example of stimuli used, and part (**B**) an example of acquisition run structure. Each run consisted of 20-speriods of one of the experimental task or its reference alternating with 10 s-periods of fixation.

The fMRI experiment included a prospective graspability judgment, a motor imagery, and a perceptual length judgment task as well as their reference tasks (Fig. 1A). The graspability judgment (GJ) required participants to judge whether they would be able to grasp a rectangle lengthways between their index finger and thumb, without actually moving their fingers. To minimize head motion, participants had to respond “yes” aloud only when they judged the rectangle as graspable but stay silent when they judged it as ungraspable. The stimuli consisted of seven black rectangles with a height of 1 cm and a length equal to the maximum grip aperture (MGA) of the participant ± 0, 1, 2, or 5 cm. The reference task was a detection task in which the participant was required to say “yes” aloud when each time a 3 × 1 cm rectangular shape appeared on the screen. The motor imagery task (MI) required participants to imagine themselves grasping an object displayed on the screen as if they were about to use it. The stimuli consisted of colored pictures of 40 everyday objects (see Supplementary Fig. 1). The reference task consisted of the display of the same pictures scrambled in such a way that objects could no longer be identified. The length judgment (LJ) required participants to judge whether a rectangle was larger or not than a standard shown just before the beginning of the acquisition run. Participants had to respond “yes” aloud when the bar was larger than the standard and stay silent when the response was “no”. The stimuli and the reference task were the same as those used for the graspability judgment, except that the stimulus length was computed from the MGA of another participant ± 0, 1, 2, or 5 cm, so that the references used for length and graspability judgments were different for each participant but equal across participants (mean ± SE: 12 ± 0.18 cm).

#### Procedure

To optimize the signal-to-noise ratio while controlling for speech-related head motion artefacts^27^, we used a block-design paradigm, with ten blocks lasting 20,000 ms each, alternating an experimental task and its reference interleaved with 10,000 ms fixation periods (Fig. 1B). Within each block and trial, each stimulus was projected on a screen, in the rear of the scanner, and viewed by the participant via a tilted mirror mounted on the head coil for 2000 ms, with a 500-ms inter-trial interval. For the graspability and length judgments, each block consisted of eight trials including three rectangles smaller than the participant's MGA/standard, three rectangles larger than their MGA/standard and two of the length of their MGA/standard, presented in a random order. For the reference (detection) task, each block consisted of 4 to 5 stimuli presented with a random inter-trial interval. For the motor imagery, each experimental block included eight objects and each reference block eight scrambled objects. Each pair of experimental and reference tasks was used in two fMRI acquisition runs, resulting in six runs which order was pseudo-randomly counterbalanced across participants such that the graspability judgment was always performed before the motor imagery task in order to keep mental simulation implicit during the graspability judgment (i.e., LJ–GJ–MI, GJ–LJ–MI and GJ–MI–LJ). Verbal responses were conveyed by a plastic tube to a digital recorder placed in an anechoic box outside the scanner room^28^. The responses were recorded and analyzed off-line to verify that participants made accurate judgments and observed the specific instructions given for each task (see Supplemental Material).

#### Imaging data acquisition

For each participant, a high-resolution anatomical image was first acquired with a 3.0 T magnetic resonance imager (Achieva, Philips Medical Systems) and an 32-channel phased array head coil using a T1-weighted 3D turbo fast field-echo sequence with an inversion recovery prepulse (time to echo [TE] = 4.6 ms, time for repetition [TR] = 9.1 ms, flip angle [FA] = 8°, field of view [FOV] = 220 × 197 mm2, 150 contiguous axial slices of 1 mm, voxel size = 0.76 × 0.76x1 mm3, SENSE factor = 1.4). Functional images were then acquired as series of blood-oxygen-sensitive T2*-weighted echo-planar volumes (GRE-EPI) with the following parameters: TE = 32 ms, TR = 2500 ms, FA = 90°, FOV = 220 × 220 mm^2^, 36 contiguous axial slices acquired in an ascending sequence, slice thickness = 3.5 mm with no interslice gap, voxel size = 1.96 × 1.96 × 3.5 mm^3^, SENSE factor (parallel imaging) = 2.5. Each acquisition run comprised 124 volumes, resulting in 80 volumes per experimental/reference task.

#### Data analysis

##### Whole-brain analysis

Brain imaging data were processed using Statistical Parametric Mapping (SPM12, Welcome Department of Imaging Neuroscience, London UK^29^). All functional volumes were (1) realigned to the first volume of the respective run (closest to the anatomical scan) to correct for within- and between-run motion, (2) coregistered with the anatomical image, (3) corrected for slice acquisition delays, (4) normalized to the standard MNI template using a Trilinear interpolation and a voxel size of 2 × 2 × 2 mm^3^, and (5) smoothed with an 5-mm FWHM Gaussian kernel. During realignment, drifts in the translation or rotation (i.e., roll, pitch, yaw) were checked for each participant and run. All data showed drifts inferior to 5 mm in translation and to three degrees in rotation. Condition-related changes in brain activity were estimated for each participant by a whole-volume voxel-wise analysis using a general linear model on which the responses evoked by each condition of interest were modeled by a standard hemodynamic response function. The contrasts computed at the individual level corresponded to each task contrasted with its reference (GJ–refGJ, MI–refMI, and LJ–refLJ). We then examined the critical contrasts at the group level. In order to reveal the brain areas commonly activated in GJ and MI, the contrasts computed at the group level were entered in a conjunction analysis ([GJ–refGJ] AND [MI–refMI]). In order to identify brain areas of this common network that were specific to the context of action, the areas found in the contrast of the length judgment and its reference were removed from the conjunction using a mask ([GJ–refGJ] AND [MI–refMI] masked exclusively by [LJ–refLJ]). We also sought for differences between the graspability and length judgment tasks by computing the following subtractions: [GJ–refGJ]–[LJ–refLJ] and [LJ–refLJ]–[GJ–refGJ]. We reported only activations surviving a statistical threshold of *p* < 0.001 corrected at the cluster level with *Random Field Theory* for *Family Wise Error* (FWE)^30,31^ and extending to at least 10 contiguous voxels.

##### Voxelwise correlations

In order to test whether the overlapping activations found in the whole-brain analysis could result from intermingled but distinct neural networks, voxelwise correlations between task-related effects were computed at the best spatial resolution achievable with our data (i.e., 8 mm^3^). The rationale is the following: if tasks A and B involve a similar pattern of activation distribution within region X, the voxels of this region that are the most (or less) activated in response to tasks A and B should be identical, leading to a positive correlation between the signal change in tasks A and B when computed across voxels of region X. Alternatively, a null or negative correlation would indicate that the voxels that are the most (or less) activated in response to task A are different than those that are the most (or less) activated in response to task B, suggesting separate but intermingled networks within region X for tasks A and B^18,19^. The clusters revealed by the conjunction analysis of the three experimental tasks were intersected with a 5-mm-radius sphere centered on peak voxels of each of the clusters. The t-values were then extracted from the resulting regions for each contrast and each participant from a set of normalized but unsmoothed data. We then computed Pearson coefficients at the individual level for the following correlations: (1) between the t-values of the [GJ−refGJ] contrast and the t-values of the [MI−refMI] contrast, (2) between the t-values of the [GJ−refGJ] contrast and the t-values of the [LJ−refLJ] contrasts and (3) between the t-values of the [MI−refMI] contrast and the t-values of the [LJ−refLJ] contrasts. To estimate whether significant correlations reflect a high degree of similarity between tasks, we also computed voxelwise correlations on the contrasts between experimental and reference tasks using a split-half procedure. This allowed us to compare the between-task correlations to the within-task correlations. Finally, in order to rule out that these correlations could be driven by physiological or measurement-related artefacts or general processes such as attention, we computed a representational similarity matrix (RSM) from the betas values and compared the pattern of correlations among experimental tasks with the pattern of correlations among reference tasks. If the correlations were driven by basic confounds, the reference tasks should also correlate with each other. The mean coefficient of each correlation was tested against 0 using a one-sample t-test and coefficients were compared to each other when relevant by using paired-sample t-tests. T-tests were corrected for multiple comparison with Bonferroni correction.

##### Lateralization

We also investigated the lateralization of the activations during graspability judgments, length judgments and motor imagery by extracting the t-values from the left vs. right aIPS for each participant and each experimental task contrasted with its reference. These regions of interest (ROIs) were defined as 5-mm-radius-spheres, centered around the average x, y, z coordinates obtained from a meta-analysis of the regions constitutive of the grasping execution network^32^, which were then transformed into the MNI space. The resulting MNI x, y, z coordinates were − 39, − 44, 44 for the left aIPS and 42, − 43, 47. The definition of the ROIs and the extraction of the t-values were performed with the MarsBAR toolbox (http://marsbar.sourceforge.net^33^). The t-values of the left and right aIPS were then compared, separately for each experimental task, using paired sample t-tests corrected for multiple comparisons with Bonferroni correction.

### TMS-MEP experiment

#### Participants

Nine healthy volunteers (7 females, *M*_age_ = 24 ± 0.94 years) participated in this experiment. They were right-handed according to the Edinburgh inventory questionnaire^26^, had no history of neurological or psychiatric disorders, had normal or corrected-to-normal vision, and had not participated in the fMRI experiment. All were screened for TMS contraindications, including recent use of alcohol, caffeine and drug^34^. They gave their written informed consent prior to the experiment and were unaware of the purpose of the study. The study was non-invasive and performed in accordance with the ethical standards of the Declaration of Helsinki.

#### Tasks and stimuli

Participants were required to perform similar prospective graspability judgments and length judgments as in the fMRI experiment, with two main changes. First, in order to disentangle the veridical size of the graspable stimuli from their perceived size relative to the visual context, judgments were performed on a horizontal line ended by arrows pointing inwards or outwards. Typically, when the line is ended by inwards pointing arrows (> <), it appears to be longer, while it appears smaller when it is ended by outwards pointing arrows (< >), corresponding to the Müller-Lyer illusion configuration. We also used an additional figure with vertical lines (| |) displayed at each end of the horizontal line as a control, not inducing any illusion. Previous studies showed that the size distortion is striking in perceptual length judgments, but its effect on grip aperture during grasping movement towards the horizontal line is significantly smaller, most probably because action is more object-centered and less influenced by the visual context than perception^35,36^. Second, in order to get a finer behavioral measure, we added two intermediate lengths for the horizontal line resulting in − 5, − 3, − 2, − 1, 0, + 1, + 2, + 3, + 5 cm, with respect to the standard (length judgment) or maximum grip aperture (graspability judgment). Mean stimuli length was 11 ± 0.41 cm for both the graspability and length judgments. The analysis of behavioral performance showed that participants performed the two tasks correctly while relying on the veridical size of the stimuli to judge their graspability and on the perceived size to judge their length (see Supplemental Material).

#### Experimental procedure

The graspability and length judgments were performed on distinct sessions scheduled on two different days at a one-week interval. During each session, the participants sat at 60 cm of a computer screen with both hands resting on a table, palms down and arms semiflexed. Each trial started with the presentation of a fixation cross for 500 ms followed by a Müller-Lyer figure. The participants responded “yes” or “no” aloud to indicate whether they judged the horizontal line as graspable or not (graspability judgment) or as larger than the standard or not (length judgment). The instructions emphasized the need to respond as quickly and accurately as possible, and responses were noted by the experimenter. After response, the figure disappeared and a blank screen was displayed for a time randomly set between 3000 and 5000 ms before a new trial started. For each judgment, all possible combinations of lengths (i.e., 9 values), illusions (i.e., 3 displays) and TMS timings (i.e., 3 delays, see below) were repeated three times, in random order, giving rise to 243 trials divided in two sub-blocks.

#### TMS-MEP protocol

Single-pulse TMS was applied with a 70 mm figure-of-eight coil connected to a Magstim 200 magnetic stimulator (Magstim, Whitland, Dyfed, UK). The coil was placed over the left M1, tangentially to the skull, with the handle pointing backward and laterally at a 45° angle away from the sagittal axis, approximately perpendicular to the central sulcus^37,38^. MEPs were recorded following each TMS pulse with surface electromyography (EMG) electrodes disposed on the FDI, APB and ADM muscles of the right hand. EMG data were collected for 1000 ms spanning the range of 500 ms before to 500 ms after the pulse. The EMG signals were amplified (× 1000), bandpass filtered online (10–500 Hz; NeuroloLog; Digitimer), and digitalized at 2000 Hz for offline analysis.

In each participant, we first located the optimal spot for eliciting MEPs in the FDI muscle (*i.e.*, the “hotspot”). This site was marked on a cap fitted on the participant's head to provide a reference point of M1 throughout the experimental session^39^. The resting motor threshold (rMT) was defined as the lowest stimulation intensity allowing the generation of MEPs of at least 50 μV peak-to-peak on 5 out of 10 consecutive trials in the FDI muscle. Across participants, the rMT was 43% (± 7.4) of maximum stimulator output. The TMS intensity used during the experimental session was set at 115% of the rMT^24^. At this intensity, a single TMS pulse delivered with a figure-of-eight coil, which stimulates a zone of about 10 mm of diameter, elicits MEPs simultaneously in adjacent hand muscles (*i.e.*, in the FDI, APB and ADM muscles) as corticospinal neurons projecting to different muscles of a given limb strongly overlap within M1^39–41^. To probe the putative changes in CSE occurring during graspability and length judgments, we delivered a pulse at one out of three possible timings in the two tasks: (1) 150 ms or (2) 300 ms after stimulus onset, and (3) between 3000 and 5000 ms after response onset during the inter-trial interval (ITI). The timings of stimulation were defined after the observation of magnetoencephalography activity during the mental rotation of hand representation in the visual cortex from 150 to 180 ms after stimulus onset and in the parietal and premotor areas from 200 to 400 ms after stimulus onset^42^. The pulses at 150 and 300 ms after stimulus onset thus provide a measure of corticospinal activity before and during the period the hand representation is expected to be transformed in motor areas under the assumption that graspability judgments involves motor simulation. A total of 81 MEPs was recorded at each timing in each task, with three MEPs for each possible combination of object size and illusion display. In addition, we recorded 20 MEPs, before each sub-block started, to obtain a baseline measure of MEP amplitude throughout the experiment.

#### Data analysis

Trials with background electromyography (EMG) activity (root mean square computed from − 250 to − 50 ms before the TMS pulse) exceeding 2.5 SD above the mean were discarded (2.22% removal). This was done to prevent contamination of the MEP measurements by significant fluctuations in background EMG^37,43^. We then extracted the MEP peak-to-peak amplitude for each trial. MEP amplitudes were averaged separately for each TMS timing, each muscle, each task and each subject. MEPs with an amplitude exceeding 2.5 SD around the mean of the condition were discarded (73 ± 4 trials remaining per condition). A generalized linear mixed model (GLMM) was then used to model the MEP amplitude in each muscle, separately, with judgment (length vs. graspability), timing (baseline_,_ 150 ms, 300 ms vs. ITI) and their interaction as fixed effects, and the differences between participants as random effects. Bonferroni correction was applied to post hoc comparisons where relevant.

## Results

### fMRI experiment

#### Whole-brain analysis

##### Graspability judgment

**Figure 2:**
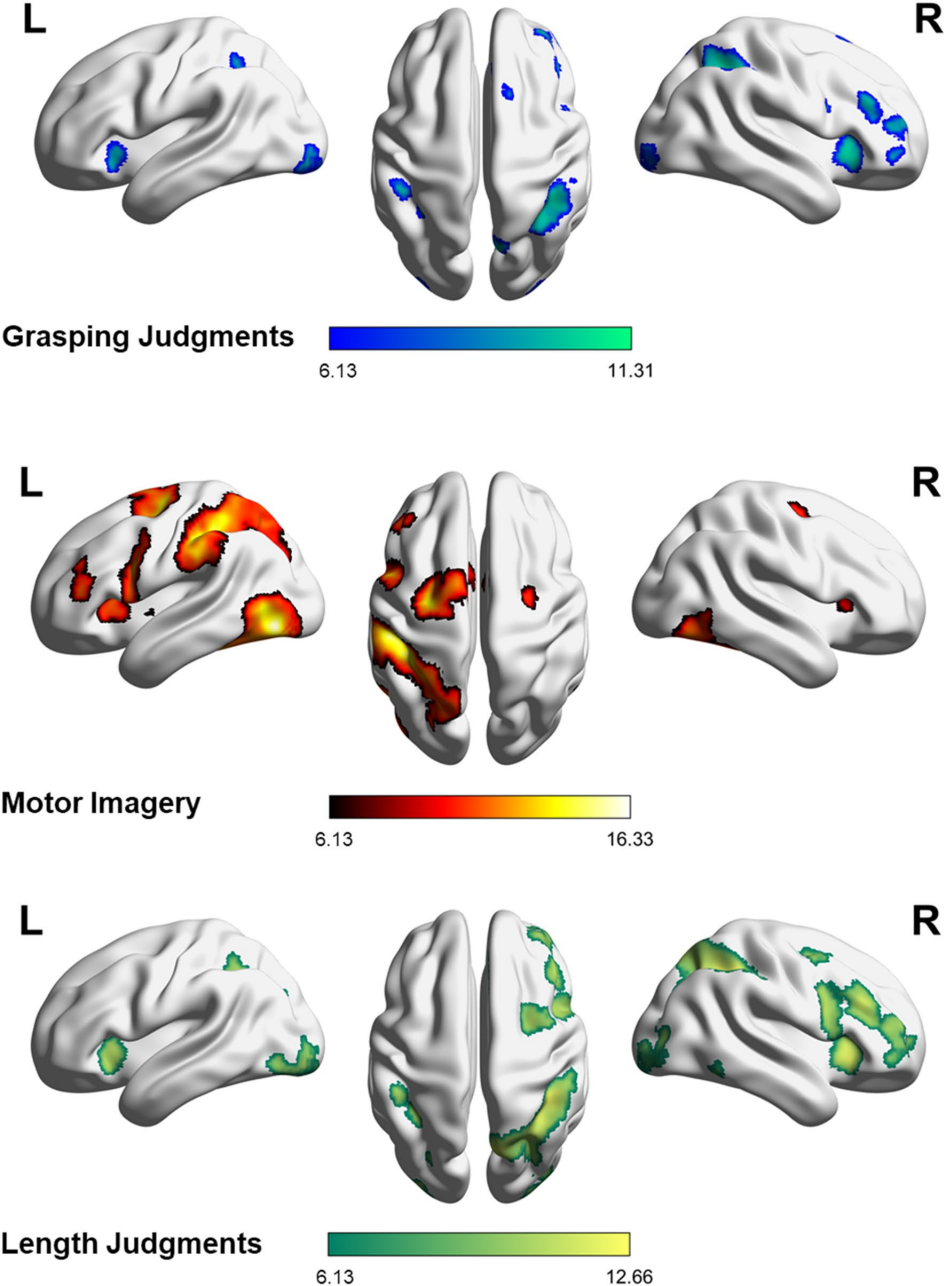
Rendering of the statistical maps showing the cortical areas activated in (**A**) the graspability judgment task vs. its reference, (**B**) the motor imagery task vs. its reference and (**C**) the length judgment task vs. its reference, surviving at a statistical threshold of *p*_*FWE*_ < 0.001 corrected at the cluster level for multiple comparisons and extending to *k* = at least 10 contiguous voxels^53^.

Contrasting fMRI responses for the graspability judgment task with those for its reference task revealed brain activations bilaterally in the inferior parietal lobule (IPL) and along the anterior–posterior axis of the IPS, including the aIPS (MNI coordinates x, y, z: 42, − 49, 47 and − 45, − 37, 44). Parietal activations in the right hemisphere included the supramarginal and angular gyri, the superior parietal lobule (SPL) and extended to the precuneus. Frontal activations were almost restricted to the right hemisphere, including the right middle and anterior cingulate cortex and the superior and middle frontal gyri in the dorsolateral prefrontal cortex (DLPFC) and the SMA. Activations were also registered within the pars opercularis, orbitalis and triangularis of the right inferior frontal gyrus, which partly corresponded to the rostral part of PMv. In the occipital areas, clusters of activation were found around the right calcarine sulcus and in the left inferior occipital gyrus, corresponding to secondary (V2) visual areas. Other clusters of activations were found in the left posterior lobe of the cerebellum (VI and crus I and II of VII), and in the left and right insula (Table 1a and Fig. 2).

**Table 1:** Brain regions showing significant activations for (a) graspability judgments, (b) motor imagery and (c) length judgments compared to their own reference task. k = cluster size (number of voxels); x,y,z = peak coordinates (MNI); T = t-statistic.

	Anatomical regions	*k*	*x*	*y*	*z*	T
**a**	**[GJ−refGJ]**					
	Right calcarine sulcus	340	18	− 90	− 2	11.31
	Right middle cingulate gyrus, medial superior frontal gyrus	387	6	26	38	10.57
	Left inferior occipital gyrus	232	− 24	− 94	− 10	10.29
	Right insula	365	36	20	2	10.13
	Right inferior parietal lobule, intraparietal sulcus, superior parietal lobule	811	38	− 56	48	9.29
	Right middle frontal gyrus	139	42	50	12	8.71
	Right precuneus	71	8	− 70	52	8.45
	Right middle frontal gyrus and triangular part of the inferior frontal gyrus	152	46	32	28	8.33
	Left inferior parietal lobule, intraparietal sulcus	49	− 46	− 38	44	8.00
	Left cerebellar lobule VI and VII (Crus I)	80	− 32	− 66	− 30	7.76
	Left cerebellar lobule VII (Crus II)	24	− 6	− 78	− 26	7.46
	Left insula	89	− 34	18	− 2	7.44
	Right superior frontal gyrus, bilateral supplementary motor area	18	14	20	64	7.40
	Right orbital part of the right inferior frontal gyrus	25	44	46	− 4	7.06
	Left inferior parietal lobule	31	− 38	− 50	50	6.88
	Right supramarginal gyrus	10	52	− 32	48	6.72
	Right anterior cingulate gyrus	10	8	36	22	6.69
	Right precentral, opercular part of the frontal inferior gyrus	20	44	4	26	6.50
**b**	**[MI−refMI]**					
	Right cerebellar lobule VI and VII (Crus I), inferior occipital gyrus, inferior temporal gyrus	1370	34	− 52	− 28	16.34
	Left inferior occipital gyrus, cerebellar lobule VI and VII (crus I), fusiform gyrus	2425	− 44	− 74	− 8	16.05
	Left inferior parietal lobule, intraparietal sulcus, superior parietal lobule, precuneus, middle occipital gyrus	3474	− 50	− 28	42	14.69
	Left precentral gyrus, anterior cingulate gyrus, bilateral supplementary motor area, left middle frontal gyrus	2111	− 26	− 12	56	14.55
	Left precentral gyrus, opercular part of the inferior frontal gyrus, insula	1176	− 52	8	26	12.73
	Left middle frontal gyrus, triangular part of the inferior frontal gyrus	243	− 44	38	12	8.94
	Right precentral, superior frontal gyrus	102	26	− 10	56	7.81
	Right insula, opercular part of the inferior frontal gyrus	40	36	20	4	7.27
	Left insula	18	− 40	0	2	6.81
	Left putamen	22	− 20	2	6	5.96
**c**	**[ LJ−refLJ]**					
	Right inferior parietal lobule, including the angular and supramarginal gyri, intraparietal sulcus, superior parietal lobule, precuneus, middle occipital gyrus	2616	40	− 42	44	12.66
	Right insula, triangular, orbital and opercular parts of the inferior frontal gyrus, middle frontal gyrus	2242	48	30	28	11.87
	Right middle and anterior cingulate gyrus, medial superior frontal gyrus	867	6	26	38	11.85
	Left cerebellar lobule VII (Crus I)	423	− 32	− 66	− 30	11.42
	Right calcarine sulcus, inferior and middle occipital gyri	490	18	− 92	− 2	11.26
	Left cerebellar lobule VII (Crus II)	189	− 6	− 78	− 28	10.38
	Left inferior occipital gyrus, calcarine fissure, fusiform gyrus	348	− 24	− 94	− 10	10.30
	Left insula	290	− 34	18	0	9.66
	Right middle and superior frontal gyrus	448	30	2	52	9.53
	Left intraparietal sulcus	83	− 46	− 38	44	8.85
	Left inferior parietal lobule	148	− 34	− 52	46	8.41
	Left middle occipital gyrus	37	− 26	− 74	28	7.95
	Right inferior temporal gyrus	54	54	− 50	− 10	7.60
	Left inferior parietal lobule, middle occipital gyrus	10	− 26	− 60	40	6.35

##### Motor imagery

Contrasting the motor imagery task with its reference revealed a large left-lateralized fronto-parietal network. Parietal activations were found in the left IPL (including the supramarginal gyrus), along the anterior–posterior axis of the IPS (including aIPS; − 45, − 34, 41), extending to the SPL and the precuneus. Frontal activations were found in the SMA bilaterally, the left anterior cingulate cortex, precentral gyrus (in particular the PMd and caudal PMv) and middle frontal gyrus (DLPFC). Activations were also revealed in the pars triangularis and opercularis of the left inferior frontal gyrus, corresponding to rostral PMv. In the right hemisphere, frontal activations were restricted to the precentral gyrus, the superior frontal gyrus, and the pars opercularis of the inferior frontal gyrus. There were also activation foci in both the left and right associative visual cortex of the inferior occipital gyrus, the left middle occipital gyrus, fusiform area and the right inferior temporal gyrus. Finally, there were clusters of activation in the left and right posterior lobes of the cerebellum (VI and crus I of VII), the insula and the left putamen (Table 1b and Fig. 2).

##### Length judgment

Contrasting the length judgment task with its reference revealed foci of activations bilaterally in the IPL and along the anterior–posterior axis of the IPS (including aIPS; 39, − 43, 44 and − 45, 40, 44). Parietal activations in the right hemisphere included the angular and supramarginal gyri, the superior parietal lobule and the precuneus. Frontal activations were restricted to the right hemisphere and included the middle and anterior cingulate cortex, the superior and middle frontal gyri (DLPFC) and the pars opercularis, orbitalis and triangularis of the inferior frontal gyrus, which partly corresponded to the rostral part of PMv. In the occipital cortices, clusters of activations included both the right and left inferior occipital gyri and along the calcarine sulcus, and the right middle occipital gyrus, corresponding to V2 and the associative visual areas. Activations were also found in the right inferior temporal gyrus, bilaterally in the insula and the left posterior lobe of the cerebellum (VII, crus I and II; Table 1c and Fig. 2).

##### Graspability judgment and motor imagery

**Figure 3:**
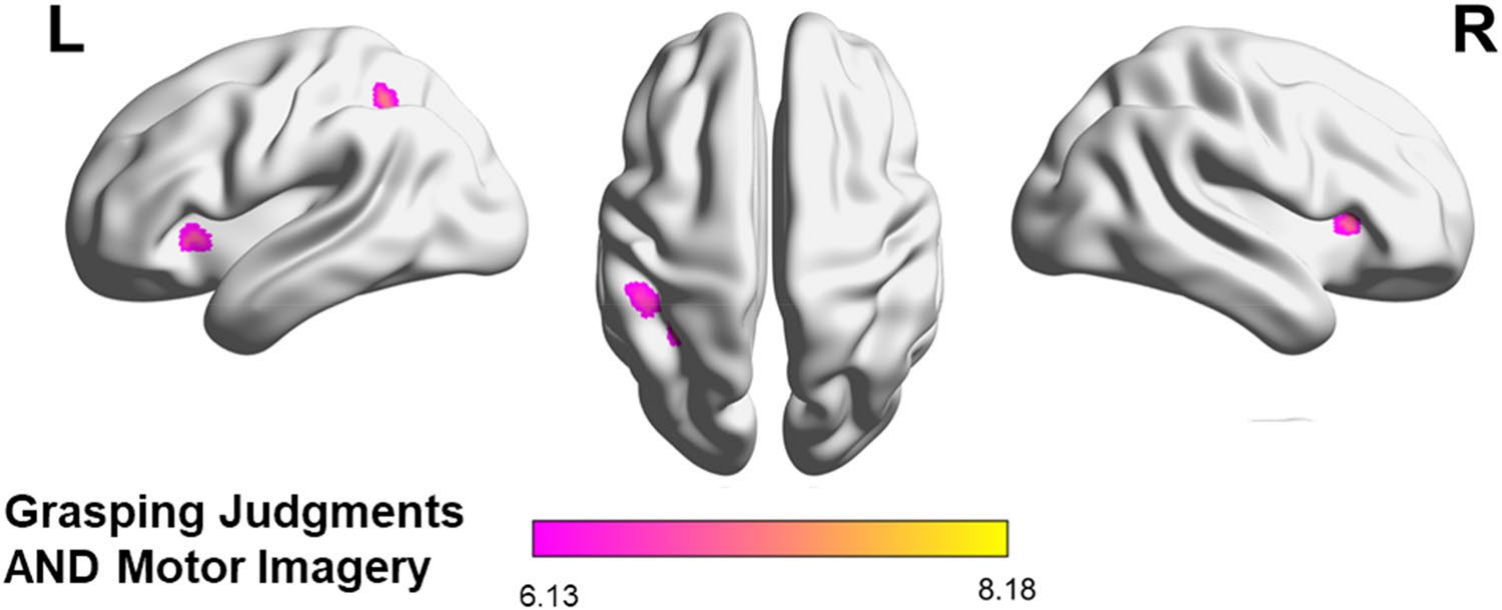
Rendering of the cortical areas commonly activated in the graspability judgment and the motor imagery task, surviving a statistical threshold of *p*_*FWE*_ < 0.001 corrected at the cluster level for multiple comparisons and extending to k = at least 10 contiguous voxels^54^.

The conjunction of graspability judgment and motor imagery, each contrasted to its own reference ([GJ−refGJ] AND [MI−refMI]), revealed the areas commonly involved in both tasks. They mainly corresponded to a fronto-parietal network including the left IPL, around the aIPS (− 45, − 37, 44), the left and right SMA and the right medial superior frontal gyrus. Additional activations were observed in the left and right insulas (Table 2 and Fig. 3). In order to identify which of these brain areas were specific to the context of action, the same conjunction was masked exclusively by the contrast between the length judgment and its reference ([GJ−refGJ] AND [MI−refMI] masked exclusively by [LJ−refLJ]). This did not reveal any significant cluster.

**Table 2:** Brain regions showing activation in (a) the conjunction of the graspability judgment and motor imagery task, (b) the contrast between graspability judgments and motor imagery, (c) the contrast between motor imagery and graspability judgments and (d) the conjunction of the graspability and length judgments, each experimental task first being contrasted to its reference. k = cluster size (number of voxels); x,y,z = MNI coordinates of peak activation at the cluster level; T = t-statistic.

Anatomical Regions	*k*	*x*	*y*	*z*	T
**[GJ−refGJ] AND [MI−refMI]**					
Right medial frontal gyrus, bilateral supplementary motor area	66	− 2	20	44	8.18
Left inferior parietal lobule, intraparietal sulcus	49	− 46	− 38	44	8.00
Right insula	25	36	20	4	7.27
Left insula	38	− 34	18	− 2	7.20
Left inferior parietal lobule	31	− 38	− 50	50	6.88

##### Graspability and length judgments

The picture emerging from the preceding task contrasts (see renders on Fig. 2) suggests that graspability and length judgments involved the same right-lateralized fronto-parietal network. To investigate further this, we contrasted graspability judgment to length judgment ([GJ−refGJ]−[LJ−refLJ]), and conversely ([LJ−refLJ]−[GJ−refGJ]). The contrasts did not reveal any difference between the brain activations elicited by the two tasks.

#### Voxelwise correlations

The conjunction analysis performed on the three tasks (*i.e*., [GJ−refGJ] AND [MI−refMI] AND [LJ−refSJ]) revealed the same 5 clusters of activation as the conjunction between graspability judgment and motor imagery (*i.e*., [GJ−refGJ] AND [MI−refMI]): SMA, left aIPS, left IPL, left insula and right insula; Table 2; Fig. 3). The voxelwise correlations on the contrast between each experimental task and its reference in each of the five regions identified by the conjunction analysis revealed positive and significant correlations in all ROIs between all pairs of tasks (all *r* ranging between 0.24 and 0.55; all *p* < 0.003). The Pearson coefficients for the correlation between the t-values of [GJ−refGJ] and [MI−refMI], [GJ−refGJ] and [LJ−refLJ] and between [MI−refMI] and [LJ−refLJ] are reported in Table 3.

**Table 3:** Mean Pearson coefficients (± S.E.) of the correlations between the patterns of activation induced by the graspability judgment, length judgment and motor imagery tasks taken in pairs within the five regions revealed by the conjunction analysis of the three experimental tasks;* p-value < .05; ** *p*-value < .01.

	GJ and MI	GJ and LJ	MI and LJ
Bilateral SMA	.37 ± .06 **	.55 ± .06**	.36 ± .07**
Left IPL	.40 ± .06**	.51 ± .05**	.28 ± .07**
Left aIPS	.51 ± .06**	.63 ± .05**	.42 ± .07**
Left Insula	.24 ± .06*	.38 ± .06**	.28 ± .08*
Right Insula	.30 ± .06**	.63 ± .05**	.34 ± .07**

We then compared the size of between-task correlations with the size of within-task correlations after splitting the data from each experimental task in two functional runs. This analysis showed that, in all ROIs, correlations between two runs of the same task were positively correlated (*r* between 0.21 and 0.67, all *p*-values < 0.001) and correlations between graspability and length judgments (*r* between 0.21 and 0.57, all *p*-values < 0.01) were as large as these within-task correlations. Paired sample t-tests showed that, for these two judgments, between-task correlations were not significantly different from within-task correlations (all *p*-values > 0.05). These results suggest that the functional overlap between the circuits involved in graspability and perceptual length judgments is similar to what could be expected based on the reproducible pattern of activations within each type of judgment. Because the motor imagery task used real object pictures (vs. rectangles) and required explicit motor imagery (vs. size comparison), we expected a difference in the size of between-task correlations and within-task correlations involving motor imagery. Indeed, correlations between GJ and MI (*r* between 0.19 and 0.38, all *p*-values < 0.01, except in the left insula where *r* between 0.06 and 0.22 were mostly *ns*) and between LJ and MI (*r* between 0.17 and 0.33, all *p*-values < 0.05) were smaller than within-task correlations between two runs of MI (*r* between 0.36 and 0.67, all *p*-values < 0.001).

**Figure 4:**
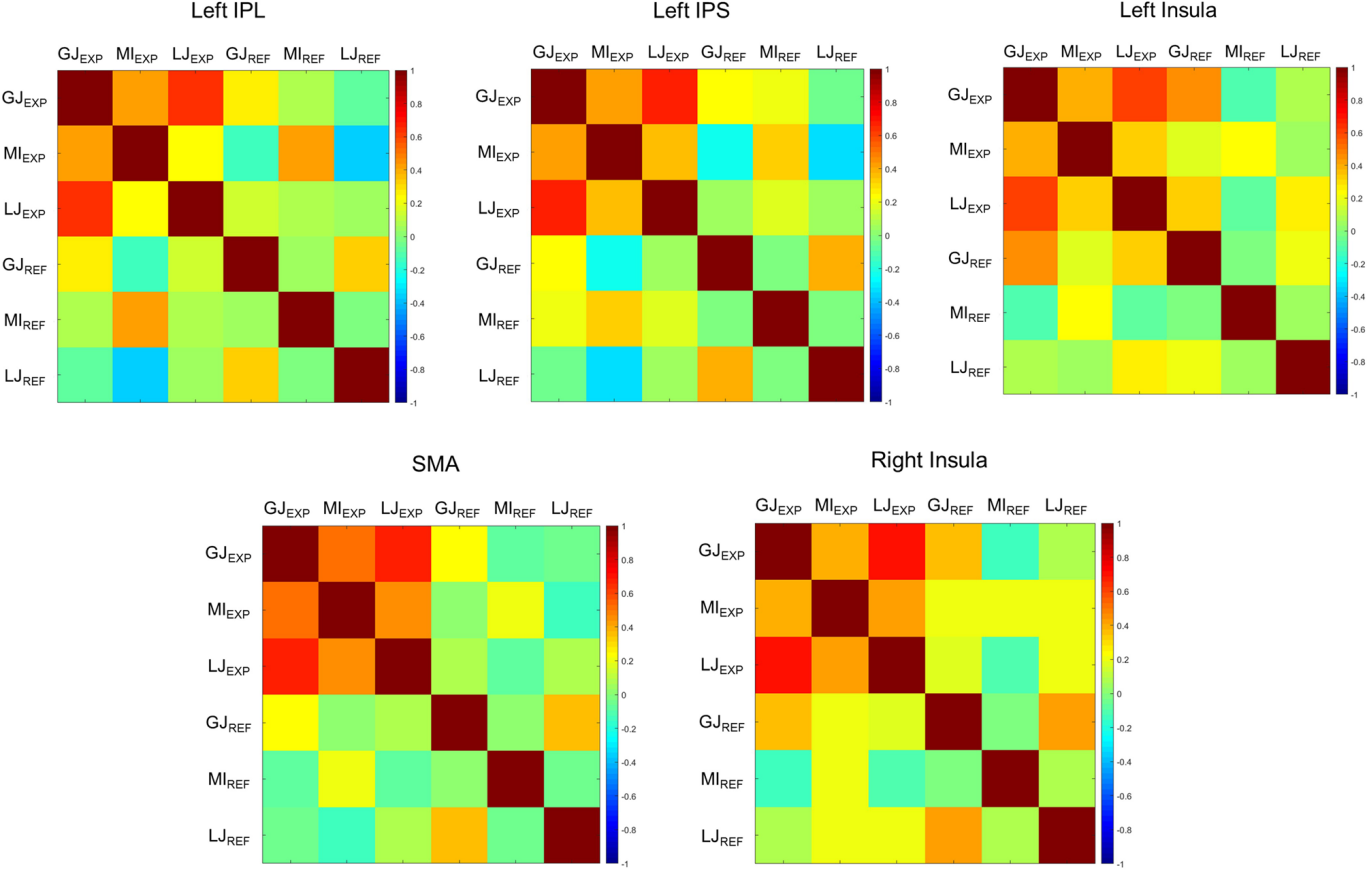
Representational similarity matrices (RSM) representing the pairwise correlations between the beta values of all experimental and reference tasks in each of the five regions commonly activated by the graspability judgment, length judgment and motor imagery tasks.

Finally, to exclude that these correlations were due to physiological or measurement-related artefacts, we extracted the beta values and computed voxelwise correlations among the experimental tasks and among the reference tasks. In all ROIs, we found positive and significant correlations between the experimental tasks (all *r* ranging between 0.24 and 0.69, all *p* < 0.001), but close to zero and non-significant correlations between different reference tasks (all *r* ranging between − 0.05 and 0.06, all *p* > 0.388). Note that the same detection task was used as a reference for graspability and length judgments explaining their positive correlation across ROIs (all *r* ranging between 0.21 and 0.42, all *p* < 0.001; Fig. 4).

#### Lateralization analysis

The comparison of the t-values extracted from the left and right aIPS indicated significantly more activation in the left compared to the right aIPS for the [MI−refMI] contrast (mean_diff_ ± SD = 1.48, *t*(29) = 8.83, *p* < 0.001). On the contrary, there was significantly less activation in the left compared to the right aIPS for the [GJ−refGJ] contrast (mean_diff_ ± SD =  − 0.47, *t*(29) = − 3.36, *p* = 0.002) and the [LJ−refLJ] contrast (mean_diff_ ± SD = − 0.88, *t*(29) = − 5.68, *p* < 0.0 01).

### TMS-MEP experiment

#### FDI

**Figure 5:**
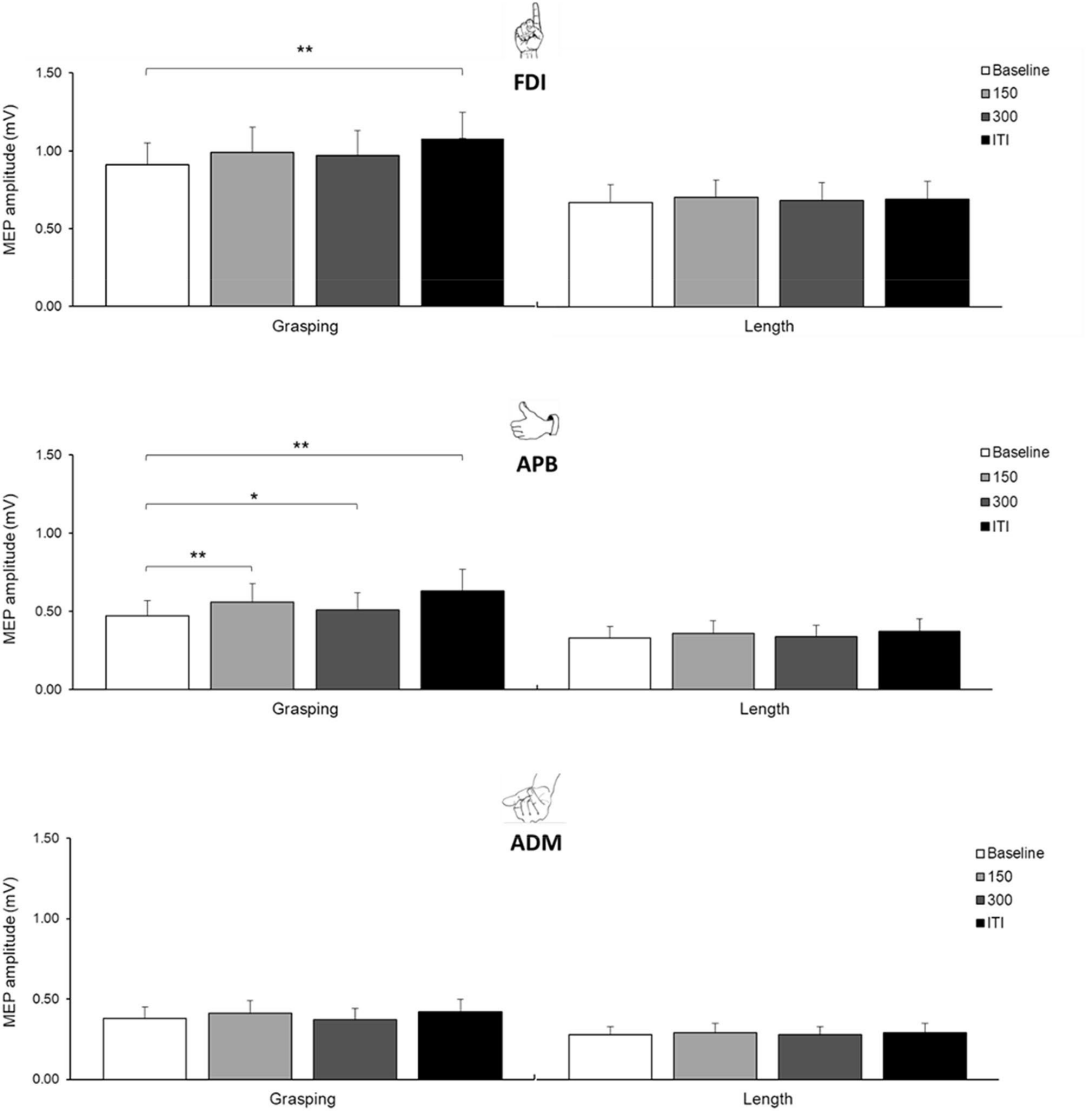
Mean MEP amplitude (mV) recorded from the right FDI, APB and ADM following TMS over left M1. MEPs are shown for the baseline out, 150 ms, 300 ms and ITI in both the graspability (left side) and length (right side) judgment. Error bar represent standard errors;* p-value < .05; ** *p*-value < .01.

The GLMM analysis on the amplitude of the MEPs recorded from the FDI showed a main effect of judgment, *F*(1,4979) = 447.69, *p* < 0.001, a main effect of timing, *F*(3, 4979) = 4.76, *p* = 0.003, and a significant interaction between the two factors, *F*(3, 4979) = 2.97, *p* = 0.031. For the graspability judgment, post-hoc pairwise contrasts indicated that, compared to the baseline (0.91 ± 0.15 mV), the MEPs were significantly larger during the ITI (1.10 ± 0.16 mV), *t*(4979) = − 3.72, *p* = 0.001. For the length judgment, post-hoc pairwise contrasts did not show any significant difference between TMS conditions (Fig. 5).

#### APB

The GLMM analysis on the amplitude of the MEPs recorded from the APB showed a significant effect of judgment, *F*(1,5076) = 565.15, *p* < 0.001, of timing, *F*(3, 5076) = 19.29, *p* < 0.001, and a significant interaction between the two factors, *F*(3, 5076) = 4.16, *p* = 0.006. For the graspability judgment, post-hoc pairwise contrasts indicated that, compared to the baseline (0.47 ± 0.10 mV), the MEPs were significantly larger at 150 ms (0.56 ± 0.12 mV), *t*(5076) = − 3.33, *p* = 0.004, 300 ms (0.51 ± 0.11 mV), *t*(5076) =  − 1.97, *p* = 0.049, and during ITI (0.62 ± 0.14 mV), *t*(5076) = − 3.92, *p* = 0.001. For the length judgment, post-hoc pairwise contrasts did not show any significant difference between the timings (Fig. 5).

#### ADM

The GLMM analysis on the amplitude of the MEPs recorded from the ADM showed a significant effect of judgment, *F*(1,4784) = 239.11, *p* < 0.001, indicating that MEPs were larger during the graspability judgment (0.39 ± 0.07 mV) than during the length judgment (0.29 ± 0.05 mV). There was a main effect of timing, suggesting an increase of MEP amplitude at 300 ms and during ITI, but pairwise contrasts with the baseline showed no significant differences (*p*-values > 0.1). There was no significant judgment x timing interaction, *F*(3, 4784) = 1.07, *p* = 0.362 (Fig. 5).

## Discussion

Previous studies suggest that predicting the outcome of an action implies the mental simulation of this action. The brain circuits supporting such prospective action judgments and motor imagery are thus expected to overlap in the parietal and frontal cortices. However, the existence and precise nature of this neural overlap has never been addressed directly by comparing the brain activations elicited by the two tasks. Moreover, part of the neural network involved in prospective action judgments is also involved in spatial cognition tasks not related to action, such as comparing two lengths^14–16^. The fronto-parietal activations observed during graspability judgments might thus indicate that they are achieved through the direct comparison of magnitude estimates, computed from grip aperture and object size, without implying any form of motor simulation as assumed earlier^1^. To disentangle these accounts, an fMRI experiment assessed the possible neural overlap of prospective judgments and explicit imagery of grasping, and tested its specificity relative to the neural correlates of perceptual length judgments. The results showed that graspability judgment and motor imagery show overlapping activations within fronto-parietal areas, but that this shared network is also common to length judgment. In all the identified fronto-parietal areas, the pattern of activation selective to each experimental task correlated positively across voxels, indicating a similar spatial distribution of activations among the three tasks. When looking more closely at the left and right aIPS, a ROI analysis revealed hemispheric specialization, with the left aIPS contributing significantly more to motor imagery than to graspability/length judgments and the right aIPS contributing significantly more to graspability/length judgments than to motor imagery. Finally, an independent TMS-MEP experiment investigated motor excitability changes at the muscle level during graspability and length judgments. The results showed that graspability judgments caused a steady increase of the CSE, in-between trials, in the hand muscles specific to the precision grip.

To sum up, the conjunction of the activations registered during graspability judgment and motor imagery revealed a fronto-parietal network including the left IPL, aIPS, and the SMA bilaterally, with additional bilateral activations within the insula. However, all these areas were also involved in perceptual length judgments devoid of any motor content. The voxelwise correlation analysis indicates, at the best spatial resolution achievable in the present study, that overlapping activations do not result from separate intermingled networks since the distribution of the activations elicited by each task was similar across voxels. Further analyses allowed us to exclude that these correlations were driven by physiological artefacts, low-level perceptual or response preparation processes. We also showed that the correlations between graspability and length judgments did not differ in size from the within-task correlations estimated from separate runs of the same task, suggesting the pattern of activation was highly similar between these two judgments. Together, these results show that the fronto-parietal network shared by the graspability judgment and motor imagery task does not dissociate from the network involved in a perceptual length judgment with no motor component, and thus cannot be interpreted as reflecting motor simulation. Instead, this network might reflect the processing of magnitude, which was a common principle of the three tasks that all required the computation of object size and/or grip aperture estimates. Previous studies have shown that areas around the IPS, in particular, are activated whenever two magnitudes presented visually (*e.g.,* sizes, lengths or orientations) have to be compared^14–16^. This recurrent finding has been taken as evidence for the existence of a generalized magnitude system housed in the horizontal part of the IPS and involved in the sensorimotor transformations achieved by the parietal cortex, such as matching grip aperture with object size^17^. This close connection between magnitude and action processing might also explain the involvement of some frontal areas contributing to graspability and length judgments. Previous studies indicated that the premotor cortex, including PMv and PMd, is activated whenever spatial distances are compared^14,15^ or held in short-term memory^44,45^. We propose that overlapping activations within the fronto-parietal cortex underlie the computation of object size estimates and the comparison to a reference maintained in short-term memory. Current models do not allow us to make precise predictions about which processes and/or representations are behind the comparison of object size and grasping capability^17,46,47^. For instance, it is not clear whether magnitude processing or stimulus size representation is behind the involvement of the parietal cortex. A former parametric study of perceptual judgments has shown that the modulatory effect of size illusions on right parietal activity depends on whether the task explicitly requires object size comparison, suggesting that active magnitude processing rather than stimulus representation might cause parietal activation^48^. These questions require further theoretical elaboration and experimental investigation using more sophisticated approaches such as representational similarity analyses or classification methods taking into account the variations in object size and the motor content of the judgments^49,50^. The critical finding of the present experiment is that fronto-parietal regions contribute to magnitude processing irrespective of body capabilities, favoring the *comparison-without-simulation* hypothesis over the *motor simulation* hypothesis.

While the results of the fMRI experiment could not dissociate the network involved in the graspability judgment from the network involved in the perceptual length judgment, the results of the TMS-MEP study revealed an increase in hand motor excitability during graspability judgment only. More particularly, graspability judgment induced a selective increase of MEP amplitude in the FDI and APB—but not ADM—muscles of the right hand. Such modulation of MEP amplitude was not observed in perceptual length judgment, which was fully matched with graspability judgment, except for the fact that it did not require activating grip representation. Thus, this increase can be viewed as the signature of the sensorimotor representation of the precision grip, which is involved in graspability, but not length judgments, and requires FDI and APB—but not the ADM muscle. However, the activation of this sensorimotor representation was not limited to the time where the judgment took place. The timing of CSE changes in FDI and APB was not locked to the resolution of the graspability judgment. Compared to baseline, the MEP amplitude increased not only during the trials, *i.e.* 150 and 300 ms after stimulus onset, but also in-between trials, *i.e.* more than 3000 ms after response onset, and this increase was actually larger in the latter condition. This pattern of results deviates from what has been previously observed in motor imagery tasks, where CSE changes are strictly bound to the progression of the imagined movement^25,51^. In accordance with the fMRI results, the MEP results do not provide evidence for graspability judgments to involve the simulation of any reach and grasp movement. The particular enhancement of motor excitability in-between trials rather suggests that participants covertly activated the motor representation of their precision grip in preparation for the upcoming trial, or in reminiscence of the previous one, as if they were refreshing their memory of the maximal achievable grip aperture to facilitate the next judgment or verify the previous judgment. While it is noteworthy that this motor representation was maintained active during the judgment, as evidenced by the lower but significant enhancement of motor excitability during each trial, the results clearly emphasize the off-line involvement of the hand motor representation whose supportive role becomes manifest in-between trials.

These findings highlight the advantage of using TMS to probe the state of the motor system in the covert stages of action. In motor imagery tasks, the BOLD signal reflecting M1 activity is indeed cut down by 30–50%, which makes it difficult to detect^20,21^, although emerging techniques such as laminar fMRI might definitely overcome this difficulty in the near future^22^. The TMS-MEP study allowed probing the motor system differently, offering a temporal resolution near 100 ms, a greater sensitivity through the direct stimulation of hand muscles, and specificity through a distinction between agonist and non-agonist muscles of the imagined movement.

The brain imaging results further showed that the fronto-parietal activations were mainly left-lateralized during motor imagery and right lateralized during graspability and length judgments. More particularly, the motor imagery task engaged the left IPL, aIPS, and SPL, the left PMv and PMd, as well as the left M1 and S1, in line with previous studies^12^. In the right hemisphere, the IPL, the rostral PMv and the SMA were also involved, though to a lesser extent. On the contrary, the graspability and length judgments showed large clusters of activations in the right hemisphere, namely the right precuneus, IPL, aIPS and SPL, as well as the SMA, right PMv and DLPFC, while activation in the left hemisphere were restricted to the IPL and aIPS. This hemipsheric lateralization was corroborated by the ROI analysis of the grasping network, with the left aIPS contributing exclusively to motor imagery and the right aIPS contributing to graspability/length judgments. The larger involvement of the left hemisphere during motor imagery is consistent with the dominant role of this hemisphere in motor functions^52,53^. The larger involvement of the right hemisphere during graspability and length judgment is consistent with the role of this hemisphere in spatial cognition and in non-symbolic magnitude comparison^14–16,46^.

It might be argued that the absence of difference between the networks involved in graspability and length judgments results from participants confounding perceptual and motor strategies. However, the instructions for the graspability and length judgments were formulated differently in order to prevent participants from adopting a similar strategy in both tasks: while participants had to judge whether a rectangle was small enough to be grasped between finger and thumb in the graspability judgment, they had to decide whether a rectangle was larger than a memorized reference in the length judgment. Hence, graspability judgments required a “yes” response to small rectangles whereas length judgments required a “no” response to small rectangles. Moreover, the perceptual reference used in the length judgment was of a different length than the MGA in each participant. Behavioral data (procedure and results reported in the Supplement Material available online) of both experiments indicated that the regression of participants’ responses on stimulus length had a negative slope in graspability judgments and a positive slope in length judgments, confirming that they answered each task differently. Furthermore, in the TMS-MEP experiment, the Müller-Lyer illusion was introduced as an additional means to differentiate the processing of relative and veridical object size estimates in length and graspability judgments. We observed that graspability judgments were less affected by the Müller-Lyer illusion than perceptual judgments, in line with the idea that object-directed actions rely on a different spatial reference frame that insulates the object from the visual context. Both judgments thus integrated information relative to the visual context but the reduced effect of Müller-Lyer illusion in graspability judgments suggests the involvement of concurrent mechanisms that refined object size estimates for action calibration. This observation goes against a strict independence of the processes underlying visual perception and object-driven action, but fits with the idea that the visual context matters less when object size is matched with grip aperture rather than with a perceptual template^47^.

To conclude, our results indicate that the fronto-parietal regions involved in graspability judgments do not dissociate from those involved in length judgments devoid of any motor content, favoring the idea that size comparison—rather than motor imagery—determines their involvement. The right hemisphere contribution to graspability and length judgments contrasts with the left hemisphere contribution to explicit motor imagery and further challenges the idea that prospective action judgments involve the mental simulation of the judged action. Our brain imaging and electrophysiological data actually rather converge to suggest that the motor system is activated covertly, at a subthreshold level, to refresh the short-term memory of one’s maximal grip aperture and facilitate its comparison with object size in subsequent judgments or verify the correctness of previous judgments. Overall, the study demonstrates how neurophysiological measurements obtained with non-invasive brain stimulation may bridge the gap between cognitive and brain imaging research and thereby renew functional hypotheses about action prediction.

## Supplementary Information

Supplementary Information

## Data Availability

Anonymized fMRI and EMG data will be available from the corresponding author on reasonable request.
