# Supplementary material for: Role of the fronto-parietal cortex in prospective action judgments
Source: Sci Rep. 2021 Apr 2;11:7454. doi: 10.1038/s41598-021-86719-9 (PMC8018944; doi:10.1038/s41598-021-86719-9)
Supplement: Supplementary file 1 — Supplementary Information [file 41598_2021_86719_MOESM1_ESM.docx]

**Role of the fronto-parietal cortex in prospective action judgments: Supplemental Material**

Laurie Geers ^1^, Mauro Pesenti ^1,2^, Gerard Derosiere^2^, Julie Duque ^2^, Laurence Dricot ², Michael Andres ^1,2,*^

^1^ Psychological Sciences Research Institute, Université catholique de Louvain, Place Cardinal Mercier 10, Louvain-la-Neuve, Belgium

^2^ Institute of Neuroscience, Université catholique de Louvain, Avenue Mounier 53, Brussels, Belgium

*Correspondance should be adressed to M.A. (email : michael.andres@uclouvain.be)

**Supplemental Figure 1**


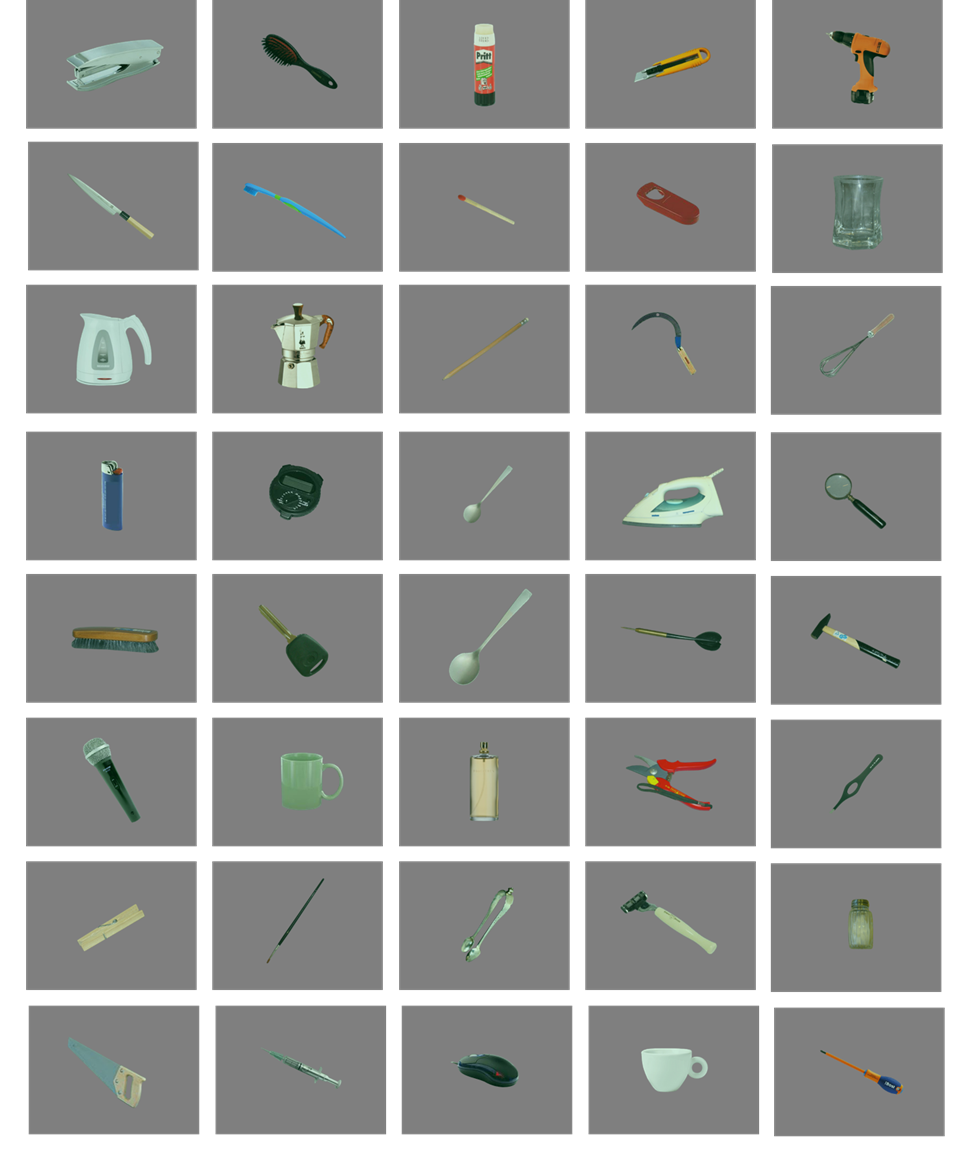


**Behavioral data analysis**

***fMRI experiment***

A generalized linear mixed model (GLMM) was used to analyze yes/no responses in a subset of 20 participants. The other participants were excluded from this analysis due to technical issues affecting the recording of their verbal responses. Because instructions associated small rectangles with a “yes” response in grasping judgments but with a “no” response in length judgments, we recoded the responses so that the length effect could be interpreted consistently in the two tasks. The “yes” response was coded 1 for the grasping judgment and 0 for the length judgment (*i.e.,* the rectangle was perceived as “graspable” and “small”), whereas the “no” response was coded 0 for the grasping judgment and 1 for the length judgment (*i.e.,* the rectangle was perceived as “non-graspable” and “large”). The GLMM included the type of judgment (GJ vs. LJ), the length of the rectangle relative to MGA/standard (-5, -2, -1, +0, +1, +2 vs. +5 cm), and their interaction as fixed effects and the differences between participants as random effects. Bonferroni correction was applied to the post-hoc comparisons where relevant.

***TMS-MEP experiment***

A GLMM was used to model the probability of yes/no responses with the type of judgment (grasping vs. length), the mli figure (short, neutral vs. long), the timing (150 ms,300 ms vs. ITI), the length of the line relative to MGA/standard (-5, -3, -2, -1, 0, +1, +2, +3 vs. +5 cm), and their interactions as fixed effects, and the differences between participants as random effects.

# Behavioral Results

## ***fMRI Experiment***

The GLMM analysis showed that length significantly predicted participants’ responses, *F*(1, 3195) = 609.28, *p* < .001, with the rate of affirmative responses decreasing for the grasping and increasing for the length judgment as the length of the rectangle increased. The analysis also revealed an effect of the judgment, *F*(1, 3195) = 29.77, *p* < .001, indicating that participants gave less positive responses for the grasping (mean ± *SE* = 0.53 ± 0.08) than for the length judgment (0.67 ± 0.08). There was also a significant judgment x length interaction, *F*(1, 3195) = 32.48, *p* < .001, indicating that the slope was steeper for the length judgment than for the grasping judgment (Supplemental Figure 2).

**Supplemental Figure 2**

**
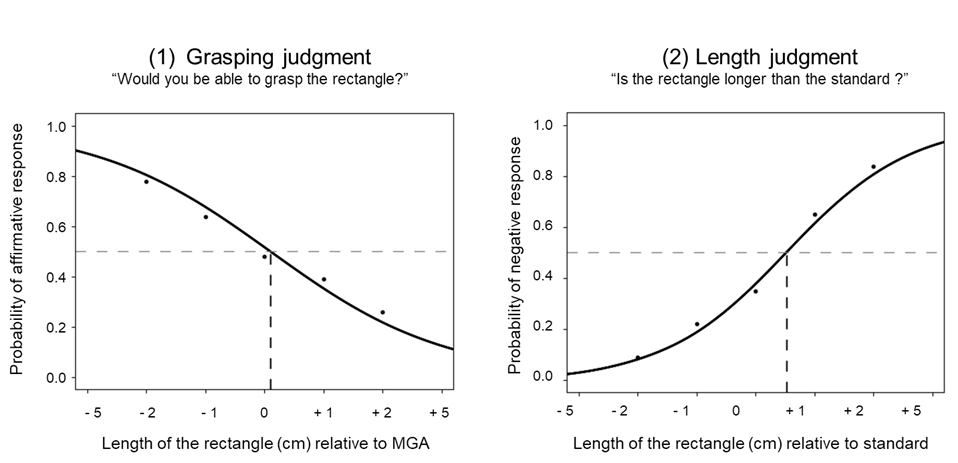
**

***TMS-MEP Experiment***

The GLMM analysis revealed a significant effect of length, *F*(1, 4346) = 798.43, *p* < .001, indicating that the rate of affirmative responses decreased for the grasping and increase for length judgments as the length of the horizontal line increased. There was also a significant effect of mli figure, *F*(2, 4346) = 186.58, *p* < .001, indicating that, compared to the neutral figure (0.73 ± 0.10), participants gave less affirmative responses to the long figure (0.15 ± 0.07), t(4355) = 9.62, p < .001, and more affirmative responses to the short figure (0.91 ± 0.04), t(4355) = -3.15, p = .002. The analysis also revealed an effect of judgment, *F*(1, 4346) = 205.88, *p* < .001, indicating that participants gave less positive responses for the grasping (mean ± *SE*: 0.34 ± 0.01) than for the length judgments (0.85 ± 0.08). There was also a significant mli x judgment, *F*(2, 4346) = 12.30, *p* < .001, and length x mli x judgment interaction, *F*(4, 4346) = 7.10, *p* < .001, indicating that the effect of the illusion was weaker on grasping than on length judgments (Supplemental Figure 3).

**Supplemental Figure 3**


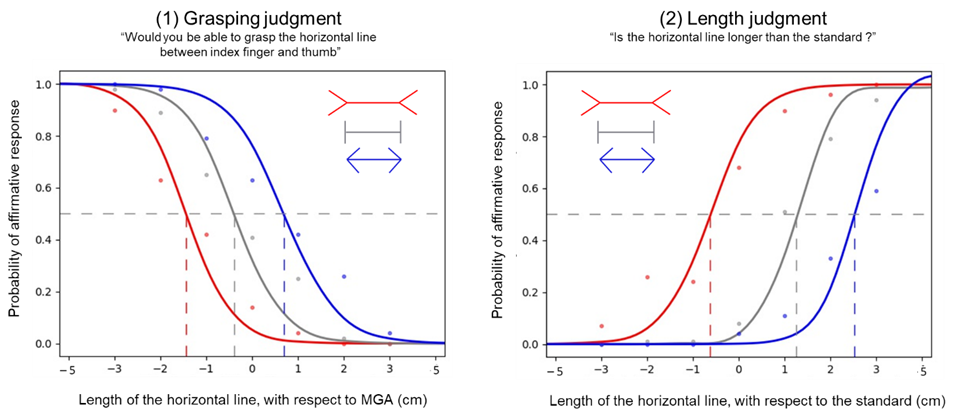


# Figures and tables legends

**Supplemental Figure 1.** Stimuli used in the motor imagery task of the fMRI experiment consisting of 40 color pictures of graspable objects.

**Supplemental Figure 2.** Fitted curves from cumulative data of size on graspability and length judgments of the fMRI experiment. The dots represent the average rate of affirmative responses (y-axis) as a function of the length of the rectangle (x-axis) with respect to (1) the maximum grip aperture or (2) the size of the standard. The solid lines represent the predicted values obtained by means of logistic regression.

**Supplemental Figure 3.** Fitted curves from cumulative data showing of the effect of the Müller-Lyer illusion on graspability and length judgments of the TMS experiment. The dots represent the average rate of affirmative responses (y-axis) as a function of the length of the rectangle (x-axis) with respect to (1) the maximum grip aperture or (2) the length of the standard. The solid lines represent the predicted values obtained by means of logistic regression.
